# Quantitative estimation of nanoparticle/substrate adhesion by atomic force microscopy

**DOI:** 10.3762/bjnano.17.1

**Published:** 2026-01-02

**Authors:** Aydan Çiçek, Markus Kratzer, Christian Teichert, Christian Mitterer

**Affiliations:** 1 Department of Materials Science, Montanuniversität Leoben, Franz-Josef-Straße 18, 8700 Leoben, Austria; 2 Chair of Physics, Department Physics, Mechanics and Electrical Engineering, Montanuniversität Leoben, Franz-Josef-Straße 18, 8700, Leoben, Austria

**Keywords:** adhesion, atomic force microscopy, magnetron sputtering, nanomanipulation, nanoparticles

## Abstract

Understanding nanoparticle adhesion to substrates is the key for their stability and performance in many applications, including energy systems, nanofabrication, catalysis, and electronic devices. In this study, we present a methodology for examining adhesion of copper nanoparticles to silicon substrates deposited under varying conditions using DC magnetron sputter inert gas condensation. Atomic force microscopy was utilized as a tool for the manipulation of the nanoparticles and to measure lateral forces for their displacement, with cantilever calibration achieved through wedge and diamagnetic lateral force calibrator methods. The work of adhesion was quantified by integrating the obtained lateral forces over the distance moved during manipulation, revealing a non-monotonic dependency on nanoparticle size with maximum adhesion observed for particles between 6 and 12 nm. In addition, an applied positive substrate bias voltage led to more energetic landing conditions and thus to increased adhesion forces. This study underscores the suitability of atomic force microscopy in characterizing adhesion on the nanoscale and offers insights into future strategies for tailoring nanoparticle/substrate interactions.

## Introduction

Nanoparticles (NPs) are at the forefront of basic research and technological innovation, captivating researchers and engineers from various fields such as energy storage [1], electronics [2], and catalysis [3]. These tiny particles, with sizes typically ranging from 1 to 100 nm, have fundamentally different properties compared to their bulk counterparts because of their large surface-to-volume ratio [4], as well as unique electronic [5] and physicochemical properties [6]. Among these properties, particle adhesion (which is determined by the interaction between the NP and the substrate) and the interface formed between NPs and substrate [7–8] play a decisive role. Particularly when the contact area between NPs and the substrate gets large compared to their volume, adhesion forces become predominant. Understanding the adhesion of NPs is expected to provide significant benefits in many applications [9–10]. A prerequisite for their application is the ability to measure and to understand their adhesion to suitable support materials. Low adhesion could be beneficial for movable parts within micro- and nanoelectromechanical systems to eliminate undesired sticking or friction [9]. In contrast, when NPs need to withstand external forces and/or harsh conditions without detachment, for example, in catalytic applications where NPs are immobilized on supports like carbon-based materials or TiO_2_ to prevent aggregation and to maintain catalytic activity, strong adhesion is required [11]. To improve adhesion, Au NPs have been stabilized on SiO_2_ substrates by embedding them into an organometallic layer, effectively immobilizing the NPs and significantly enhancing their interfacial adhesion. Their mechanical stability was tested utilizing scanning probe microscopy nanomanipulation [12]. Another approach tested for SiO_2_ NPs was functionalizing their surfaces for improved adhesion on epoxy film-covered substrate surfaces [13].

In order to develop strategies for improved NP functionality and performance, it is necessary to measure and to quantify their adhesion to the corresponding substrates. With the available highly sensitive force sensors, atomic force microscopy (AFM) is well suited for determining the adhesion between individual NPs and the supporting substrate. Applying controlled forces to manipulate NPs enables precise quantification of adhesion forces [14]. Significant progress in AFM manipulating nanometer-scale objects has been achieved, particularly in the last two decades, enhancing its capabilities and accuracy [15–17]. However, achieving consistently accurate manipulation of NPs has inherent limitations due to limited knowledge of the exact geometry of the AFM tip as well as the complex interactions involving surface contact area and interfacial friction between the AFM tip, NPs, and the substrate [18], similar to friction studies on thin films [19]. Therefore, proper calibration of the normal and lateral force constants of the cantilever is crucial in order to extract quantitative, accurate, reliable, and reproducible lateral force values from AFM manipulation experiments [20].

Overall, manipulating NPs of extremely small size (<20 nm) is still a challenge and consequently limits studies in this area [9,14]. The majority of studies concerning AFM-based NP manipulation focuses on establishing reproducible protocols for the creation of patterns and structures with NPs as building blocks, but often without detailing lateral forces involved in the experiments [15,21–22]. Rough estimations of the lateral forces were suggested to be two thirds of the applied normal force [23]. In order to assess adhesion properties, there are only a few studies providing quantification attempts of the lateral forces acting during AFM nanomanipulation [24–27]. Thus, there is little information available on the adhesion forces involved, which is critical for understanding the correlation between NPs’ positional stability and deposition conditions.

In this study, we have investigated the adhesion between Cu NPs, deposited using different landing conditions, and a Si substrate. Cu NPs were synthesized via magnetron sputter inert gas condensation at different applied substrate bias voltages to vary their kinetic energy during landing at the substrate, thereby influencing their adhesion properties. AFM was utilized as a tool for the manipulation of the NPs in order to determine the adhesion forces. The NPs were pushed in normal direction to the AFM cantilever’s long axis by scanning the surface with the AFM tip in contact mode. The corresponding lateral forces necessary to move NPs were determined. The lateral force constant of the AFM probe, comprising the AFM tip mounted on the cantilever, was calibrated based on the modified wedge and the diamagnetic lateral force calibrator (D-LFC) method [20]. Both qualitative and quantitative analyses of the measured force distributions are presented. To provide a reliable measure for adhesion forces, the mechanical work required to manipulate NPs was calculated by determining the area covered by the measured lateral force versus distance curves. The suggested approach provides insight into the complex interplay between the NP landing conditions and resulting adhesion forces.

## Experimental

### Synthesis of Cu nanoparticles

Before deposition, single-crystalline Si(100) wafer substrates, with a thickness of around 500 µm and covered by a native oxide, were cleaned in an ultrasonic bath for 10 min with ethanol, followed by rinsing with isopropanol. Then, the substrates were plasma-cleaned in a Diener electronic Tetra 30 system at a N_2_ pressure of 50 Pa for 20 min. Immediately after plasma cleaning, the substrates were loaded in the NP deposition chamber.

The NP deposition experiments were conducted using DC magnetron sputter inert gas condensation in a Moorfield MiniLab 125 vacuum system equipped with a Nikalyte NL-UHV NP source, described in detail in a previous study [28]. The NP source is mounted at 45° angle to the deposition chamber and consists of two components, namely, the magnetron head and the aggregation zone with an attached quadrupole mass filter (QMF), both with a diameter of 125 mm. In this study, only one of the three water-cooled magnetrons, equipped with a Cu target (Kurt J. Lesker, 99.999% purity) with 25.8 mm diameter and 3.2 mm thickness, was used. Prior to deposition, the base pressure in the deposition chamber was pumped down to 6 × 10^−7^ mbar. Ar was introduced as a sputtering gas, keeping a constant flow rate of 40 sccm. Sputtering was carried out at a constant current of 200 mA (≈70 W) applied to the target. The sputtered atoms start to form NPs and to grow in the aggregation zone, where the aggregation length was adjusted to 110 mm and the pressure within the aggregation was held constant at 0.5 mbar. The growth of NPs stops after passing through the orifice, where the pressure difference from the aggregation zone to the QMF causes rapid cooling. The QMF allows one to select charged NPs based on their mass-to-charge ratio. Since it is assumed that NPs are single-charged [5], the QMF can, on the one hand, be used for scanning the NP mass distribution and, under the common assumption of spherical shape and the theoretical density of Cu, the size distribution. On the other hand, the QMF can be also be employed for filtering NPs with desired masses. An AC voltage, *V* = ±250 V, with a frequency of 4.19 kHz, a DC voltage, *U* = +2.5 V, and a *U*/*V* ratio of 0.02 were used. In the filter mode, the QMF was set to a NP diameter of 7 nm. Then, the NPs pass through a mesh grid with +21.7 V grid bias voltage, enabling to determine the flux of the negatively charged NPs. It should be noted that also positively charged and neutral NPs contribute to the NP flux, which could not be detected by the positively charged mesh grid. Subsequently, NPs passing the QMF and the grid are deposited on the Si substrates, which are fixed on the substrate holder rotating at a continuous speed of 10 rpm. The pressure in the deposition chamber was set to 1.8 × 10^−3^ mbar. The NP deposition time was controlled using a shutter placed in front of the substrate holder. Before opening the shutter to start NP deposition on the substrates, a positive DC substrate bias voltage set to values between 0 V (grounded) and 1000 V was applied to the substrate holder to affect the NP landing conditions.

For the AFM measurements, a total of ten samples were prepared. For both wedge and D-LFC calibration, five samples were prepared with bias voltages of 0, 10, 100, 500, and 1000 V and corresponding deposition times of 60, 45, 10, 5, and 3 s, respectively. The deposition time was reduced for higher bias voltages to prevent full surface coverage as higher voltages have been found in our earlier work to increase the deposition rate [29]. To avoid significant oxidation of Cu NPs in ambient air, every sample was kept in a separate vacuum chamber until the AFM measurements.

### AFM characterization

**Figure 1:**
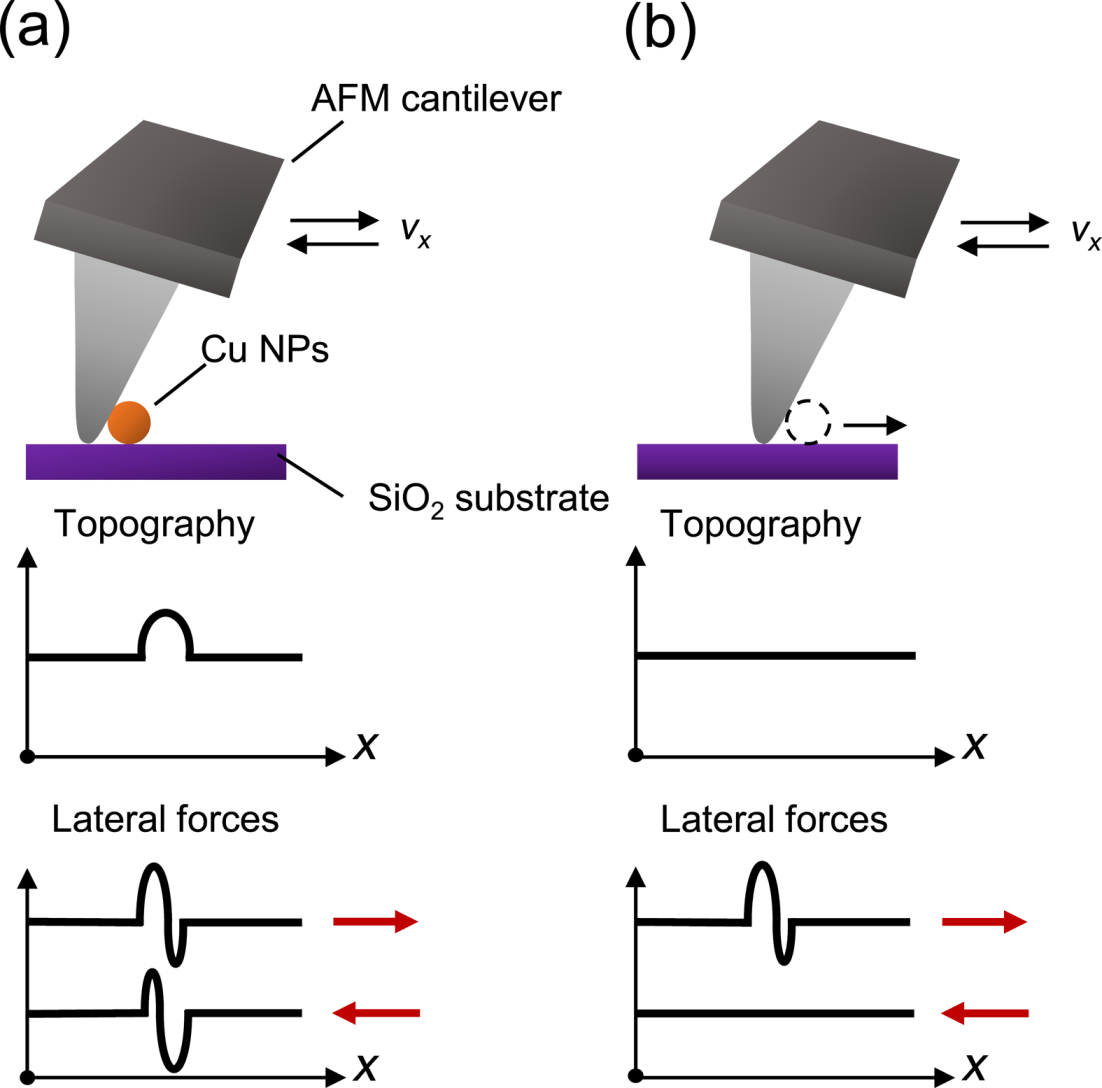
Schematics of the NP manipulation method using an AFM in contact mode. (a) The tip approaches and scans the NP when the normal force of the cantilever is low (low set point). The topography and lateral force signals are recorded during forward and backward scans. (b) As the normal force of the cantilever is adjusted to higher set points, the cantilever bending will increase. The tip comes into contact with the NP and starts to push it from its original position, that is, no corresponding topography signal of the NP can be recorded. The lateral force signal will increase when the tip makes contact with the NP to detach it and then decreases to the sliding friction force, when the tip pushes the NP along a straight line in *x*-direction. Note that, in many cases, it was found that the NP is simply pushed off from the surface without continuous sliding.

**Figure 2:**
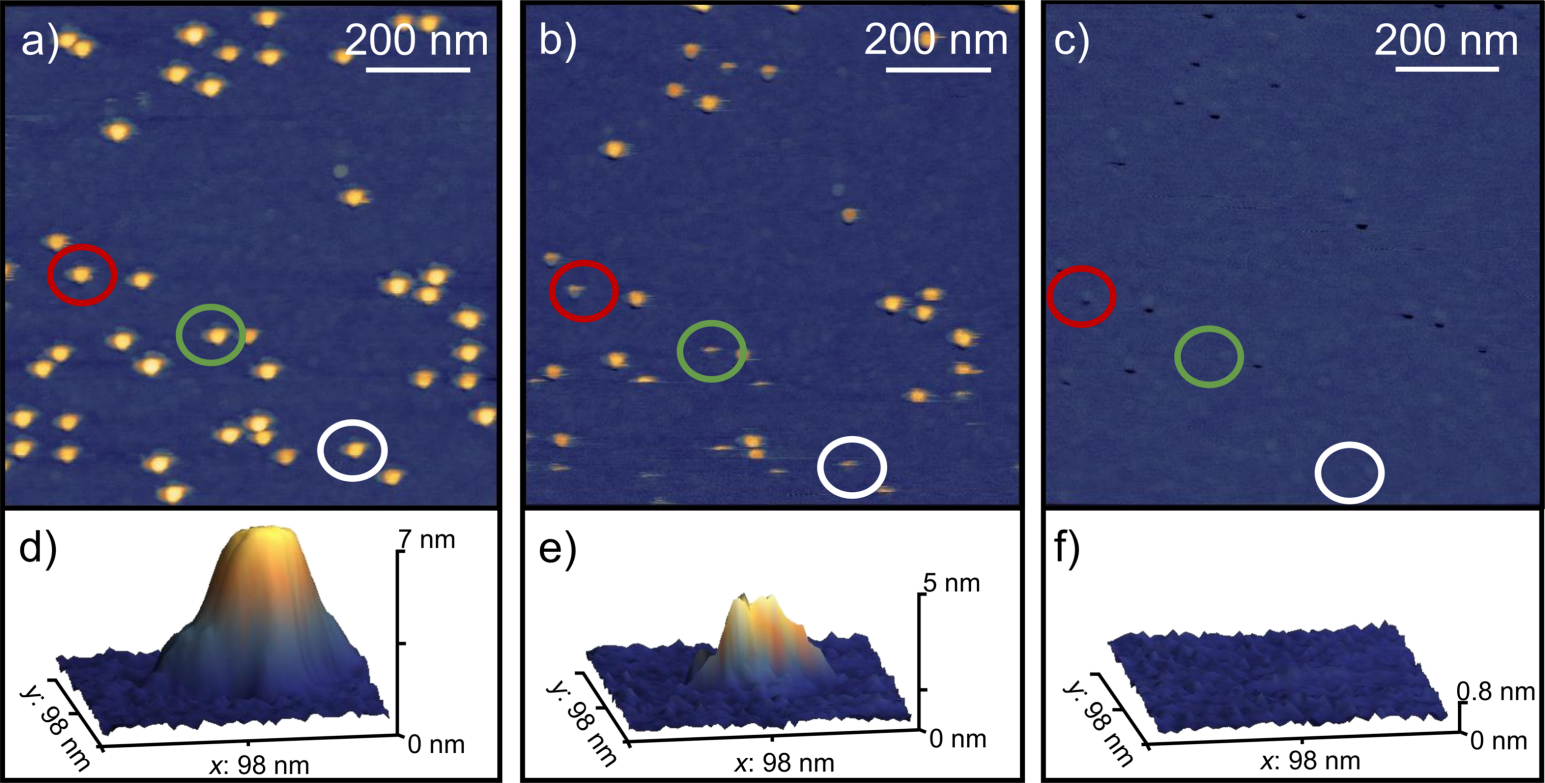
AFM topography images (1 × 1 µm^2^) of Cu NPs on Si deposited at a bias voltage of 10 V with a scan angle of 90° and a *z*-scale of 14 nm. The red, green, and white circles highlight three NP manipulation examples: (a) the initial scan for imaging Cu NPs, (b) the second scan where the NPs start to be pushed, and (c) the third scan showing the removal of NPs from the surface area. Corresponding 3D images of the green circles in (a), (b), and (c) are displayed in (d), (e), and (f), respectively. Note that the black dots in (c) are “holes” left by NPs pushed during the scanning process. A cropped AFM topography image (5 × 5 µm^2^, *z*-scale of 14 nm) of the same region, demonstrating that the displaced Cu NPs accumulate at the edges of the 1 × 1 µm^2^ scan area, is provided in Supporting Information File 1 (Figure S1).

After calibrating the cantilever by either the wedge or the D-LFC method, each sample was immediately measured at the same day. It should be noted that using a sufficiently sharp tip, the AFM investigation can proceed with the next sample without the need for recalibrating the cantilever. All AFM measurements were performed using an Asylum Research MFP 3D microscope at room temperature and under ambient conditions. AFM probes of type qp-CONT-10 provided by Nanosensors were applied in contact mode, with nominal force constants of 0.08–0.15 N·m^−1^ and tip curvature radii smaller than 10 nm. AFM topography images and lateral force data of the samples were processed using the open source software Gwyddion (version 2.63) [30]. For statistical analysis of the measured data, several independent areas were measured on each sample, ranging from 1 × 1 µm^2^ to 10 × 10 µm^2^. To obtain high-quality images, the scan speed was set to 750 nm·s^−1^ with 512 lines per frame, typically taking 30–35 min for a 10 × 10 µm^2^ image. The wedge method measurements were conducted at 60% relative humidity (RH) and a temperature of 25 °C, whereas the D-LFC method measurements were performed at 40% RH and a temperature of 18 °C. All measurements were taken with a scan angle of 90° with respect to the long axis of the cantilever, and the *z*-scale used in Gwyddion for data analysis was set to 14 nm. Note that consecutively recorded AFM images (see below in Figure 2) usually exhibit a slight thermal drift. However, this does not interfere with the data evaluation. During AFM manipulation, NPs do not always move along straight lines. Small deviations, jumps, or irregular paths can occur, depending on local variations in friction and adhesion between the particle and the surface. Rao et al. studied such behavior and showed that the stability of NP trajectories decreases with lower interfacial friction [31]. Their results support our observations that differences in adhesion and surface conditions influence how Cu NP move and detach during manipulation. Similar trajectories as observed by Rao et al. occurred very rarely in our experiments. In this study, a frequently applied standard protocol for AFM manipulation was used [32]. The protocol consists of a two-step method and is schematically illustrated in Figure 1. First, an arbitrary area with size 5 × 5 µm^2^ or 10 × 10 µm^2^ was scanned in contact mode to identify regions containing a suitable surface coverage with Cu NPs. Subsequently, more detailed scans were conducted on smaller areas of interest, that is, 1 × 1 µm^2^ or 2 × 2 µm^2^, with higher resolution. These scans captured both topography signals from vertical cantilever movement and lateral signals from twisting of the cantilever (Figure 1a). The vertical bending of the cantilever, which is linked to the height of the NPs and to their diameter (assuming spherical NPs), was recorded to provide topography images. Lateral forces were measured by observing the twisting of the cantilever during forward and backward scans. If a higher set point is chosen, that is, a larger normal force is applied to the cantilever (Figure 1b), the respective NP is pushed from its original position (as evidenced by the corresponding topography images), resulting in differences in the measured lateral forces. During NP manipulation, two primary forces dominate: The lateral force signal will increase when the tip hits the NP to overcome the NP adhesion strength to the substrate (static force) and decrease when the tip pushes the detached NP along a straight line in *x*-direction (sliding force) [16]. This approach shares similarities with manipulation of NPs from the side, known as “tip-on-side” mode [33]. However, in our approach, the tip is not pre-positioned in front of a NP of interest before manipulation; instead, it pushes NPs along the scan path on the substrate.

### Lateral force calibration methods

In order to quantify NP adhesion and sliding friction, the cantilevers used need to be calibrated. Several calibration methods for AFM lateral force have been developed [34–36]. Among these, the wedge method [37] stands out as the most commonly utilized and, with subsequently suggested modifications [38], widely accepted state-of-the-art procedure. In this study, a TGZ calibration grating from NT-MDT with defined Si slopes of 55° was used for the wedge calibration. The method is based on scanning sloped surfaces to extract lateral force calibration parameters from friction loops. However, achieving precise calibration with this indirect method requires considerable effort and extensive data processing. It relies extensively on the accuracy of the underlying mathematical models, which are based on the ratio of lateral and normal calibration constants. These ratios are obtained from the half-width and offset values of the measured torsion loops (friction loops), which are plots of lateral force vs displacement, showing frictional resistance as the tip scans a surface over a range of applied tip loads in ascending order. In the end, a series of friction loops needs to be measured for each applied tip load to calculate friction coefficients and, consequently, the lateral force calibration factor. This factor depends on the lateral sensitivity of the position-sensitive photodetector (PSPD), which gauges cantilever deflection as well as torsional spring constant [39]. However, errors can arise due to the sensitivity of the PSPD to laser alignment and a few micrometers offset from the tip shear center, leading to erroneous determination of the cantilever torsion loop offset. As a result, there is very low tolerance for measuring lateral forces with experimental errors in the nanonewton (nN) range [20]. That is why an alternative calibration method, utilizing a diamagnetic lateral force calibrator (D-LFC) [20] was developed, allowing for a direct calibration of the cantilever based on the independent calibration of the lateral force constant. In practice, this involves scanning the cantilever tip over the D-LFC surface to directly relate the deflection signal to the applied lateral force constant. Thus, the voltage signals provided by the PSPD are directly related to the lateral force applied on the tip. The sensitivity of both calibration methods is restricted by the radius of curvature of the tip, necessitating very sharp tips for the required high accuracy [20].

In this study, we calibrated the lateral force of the cantilever using either the wedge or the D-LFC method, based on the specific requirements of each experimental setup. The normal spring constant was determined using the thermal sweep method [40] implemented by default in the Asylum Research MFP-3D system. Table 1 summarizes the calibration constants and applied normal forces obtained for both wedge and D-LFC methods, which were used for all samples deposited at substrate bias voltages of 0, 10, 100, 500, and 1000 V.

**Table 1:** Calibration and manipulation parameters determined for different substrate bias voltages and calibration methods.

Substrate bias (V)	Calibration method	Lateral spring constant [N·m^−1^]	Normal spring constant (*k*_n_) [N·m^−1^]	Lateral sensitivity [nN·V^−1^]	Normal sensitivity [nm·V^−1^]	Applied normal force (*F*_n_) [nN]

0	wedge	0.1	0.1	1	89	2
10	wedge	0.1	0.1	1	89	2
100	wedge	0.1	0.1	1	57	5
500	wedge	0.1	0.5	1	57	5
1000	wedge	0.1	0.1	1	64	3
0	D-LFC	0.2	0.2	2	64	3
10	D-LFC	0.2	0.2	2	72	3
100	D-LFC	0.2	0.1	2	68	2
500	D-LFC	0.2	0.1	2	82	1
1000	D-LFC	0.2	0.1	2	57	9

## Results and Discussion

Before manipulating the NPs on each sample, surface areas of 5 × 5 µm^2^ or 10 × 10 µm^2^ were pre-scanned. A sufficiently low surface coverage of Cu NPs was defined as the criterion for choosing a suitable area of interest because it allows each NP to be pushed independently and accurately, thereby enabling precise determination of the respective lateral force. Then, within these areas, selected 1 × 1 µm^2^ areas of interest containing Cu NPs were scanned. The average lateral force required to push a Cu NP increases with increasing bias voltage, as will be discussed in this study. Indeed, at bias voltages of 10 V and below, NP pushing already during surface scanning was unavoidable under the applied measuring conditions. Even standard scanning conditions, which were chosen to be “soft” with lower set points, resulted in removal of NPs. An example is demonstrated in Figure 2; Figure 2a–c presents consecutive scans of the same surface area of Cu NPs deposited at a bias voltage of 10 V onto Si. The height (which is identical to the diameter for spherical NPs) of the Cu NP within the green circle in Figure 2a was measured to 7 nm, as evidenced by the corresponding 3D image in Figure 2d. The NP features visible in Figure 2a appear to have uniform shape and size. This indicates that the NPs are smaller than the AFM tip (tip radius ≤ 10 nm), and the tip-convolution effect [41] results in images representing rather the tip shape than the actual NPs. Figure 2b indicates that the number of NPs is reduced after each scan. The streaky features at the lower area of the image, highlighted by red, green, and white circles, represent signatures of pushing events. These streaks indicate where NPs have been displaced, vanishing from one scan line to the other. The corresponding 3D image in Figure 2e indicates a change in the NP’s position relative to the substrate. The reduction in height from 7 to 5 nm is interpreted as the initial stage of NP displacement, where the particle starts moving before the tip has reached the NP’s top. Also tilting and/or deforming of the NP might occur. After the third scan, Figure 2c and the corresponding 3D image in Figure 2f clearly evidence that all NPs were completely removed from the surface area, confirming successful pushing. The black dots in Figure 2c obviously stem from pushed NPs, leaving holes with a depth of up to 7 nm in the Si surface. The origin of these holes still needs to be clarified, but formation during NP impact (at high substrate voltages) due to plastic deformation/tilting, formation of the Cu_3_Si intermetallic phase [42], and/or fracturing of the NP/substrate interface [43] might be possible reasons. In addition, since Si(100) substrates are naturally covered by a thin native SiO_2_ layer under ambient conditions and may form a mixed SiO*_x_*N*_y_* surface after N_2_ plasma cleaning, local modification of this oxide or oxynitride layer by NP impact, such as partial penetration, removal, or reduction cannot be excluded. We did not detect changes in the roughness between pristine and plasma-treated surfaces (RMS < 0.2 nm at 5 × 5 µm area) that would contribute to a change in friction. Interfacial redox reactions between the copper NP and the wafer surface might also contribute to this behavior. However, to the best of the authors’ knowledge, no description of such reactions has been published so far. In addition, it should be noted that such pits were only observed in this specific sample.

Binns describes three distinct energy regimes for NP deposition, namely, low, medium, and high energies, corresponding to total energy per atom ranges of 0.1 eV·atom^−1^, 1–10 eV·atom^−1^, and more than 10 eV·atom^−1^, respectively [44]. In our study, size-selected Cu NPs with a diameter of 7 nm (i.e., around 15000 atoms, assuming a spherical NP shape and the theoretical density of Cu) were considered. Assuming an initial velocity of ≈100 m·s^−1^ [45] for 0 V acceleration bias, the total energy per atom increases linearly from 0.003 eV·atom^−1^ at 0 V to 0.068 eV·atom^−1^ at 1000 V, placing the single-charged NPs within the low-energy regime (soft landing) [46]. Note, the term “soft” just means that the kinetic energy carried with the NP, equally distributed over the contained atoms, is insufficient to break the metallic atom–atom bonding in the NP. However, bond breaking cannot be excluded for multiply charged and, thus, much more energetic NPs. Nevertheless, the impact can still result in high forces in the small region at the NP/substrate interface. However, under such low-energetic conditions there is a good chance that NPs deposit “softly” at their landing sites, becoming immobilized with minimal distortion and no significant surface damage.

**Figure 3:**
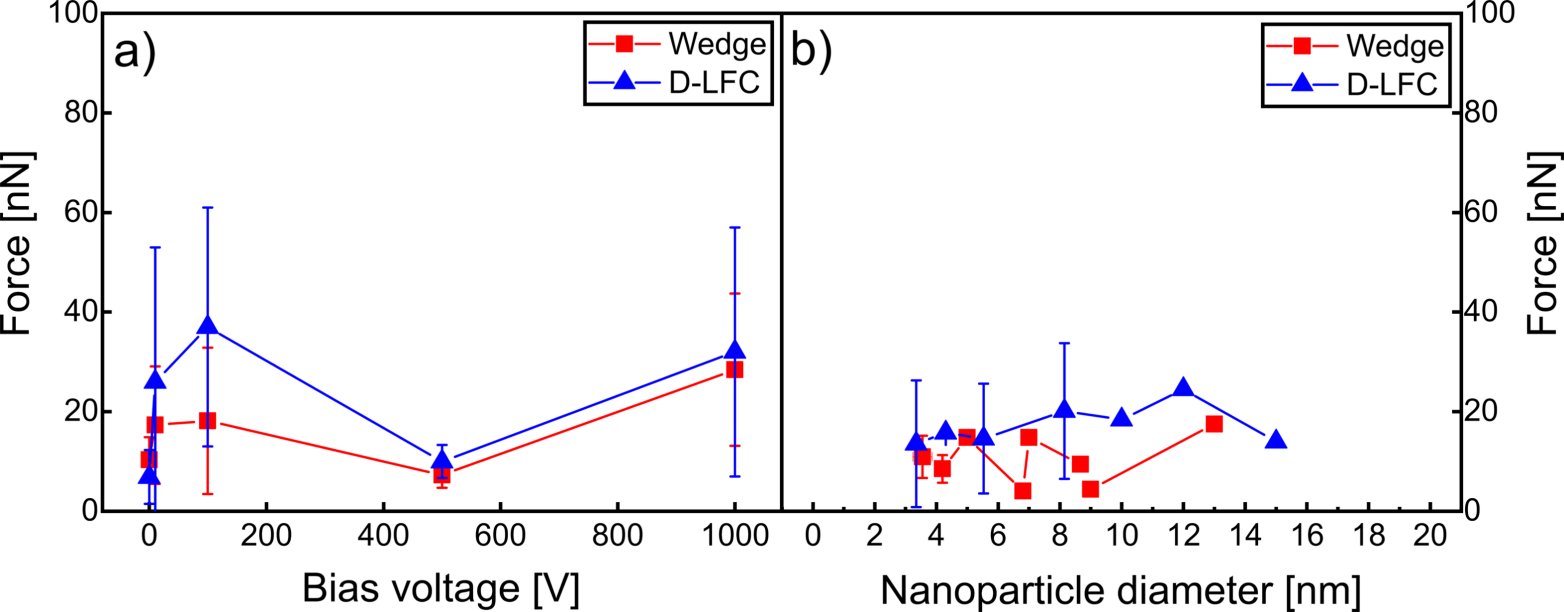
(a) Relationship between the average lateral force necessary to push Cu NPs on Si and the applied bias voltages for wedge and D-LFC calibration. (b) Average lateral force necessary to push NPs with a given diameter deposited at a bias voltage of 0 V, where the individual symbols for wedge and D-LFC calibration each include twelve data points, resulting in a total of 24 data points. Note that some error bars are too small to be visible.

Figure 3 represents an attempt to quantify the interfacial adhesion of Cu NPs on Si substrates as a function of the applied substrate bias voltage. In Figure 3a, the lateral force values required to push Cu NPs for both wedge and D-LFC methods are compared, and no clear trend emerges as all data points fall within the error bars. The significant scatter in the data, with the exception of the 0 and 500 V biases, prevents establishing a clear relationship between lateral force and substrate bias. Both calibration methods provide similar lateral force values, suggesting that measurement uncertainties are responsible for the observed scatter. Considering the results for a substrate bias voltage of 0 V in Figure 3b, the wedge and D-LFC results are closely aligned, although the wedge method yields slightly higher values. The NP size dependence of measurement accuracy might be attributed to the variations in contact geometry arising from the difference of the tip’s radius of curvature and NP diameter. Further, NP deformation and possible tip-on-NP gliding may result in additional/altered forces. The relative contribution of those additional effects is the stronger the weaker the particle adheres. Environmental factors, such as humidity and temperature, may also have contributed, particularly through enhanced capillary forces between tip and sample during AFM characterization. In general, as the NPs size increases, the lateral force required to push NPs is expected to increase. This expectation is attributed to the larger NP/substrate contact area, which strengthens interfacial adhesion forces and increases resistance to displacement. However, the interfacial forces are not only influenced by NP size but depend on the rather complex interplay of a number of parameters such as impact velocity, impact angle, surface energy, NP surface termination, relative orientation of the NP upon landing, and mechanical properties of NP and substrate [46–47]. However, a very decisive parameter is the impact velocity, which can be controlled by the substrate bias voltage applied during deposition. A higher bias voltage results in more kinetic energy of the NPs, leading to higher impact energy upon landing. The landing energy can enhance interfacial adhesion, as the NPs may embed more deeply into the substrate [29]. For larger NPs, this effect can result in even stronger adhesion; thereby, a higher lateral force is required to push them. However, plotting lateral force versus bias voltage, as done in Figure 3a, does not yield meaningful insights. Thus, we focus on the energy needed to move the NPs, as the energy is a more general and comparable parameter across different samples. Therefore, our next attempt includes the force profile and the distance along which the NPs are pushed, offering a more robust measure of interfacial adhesion.

**Figure 4:**
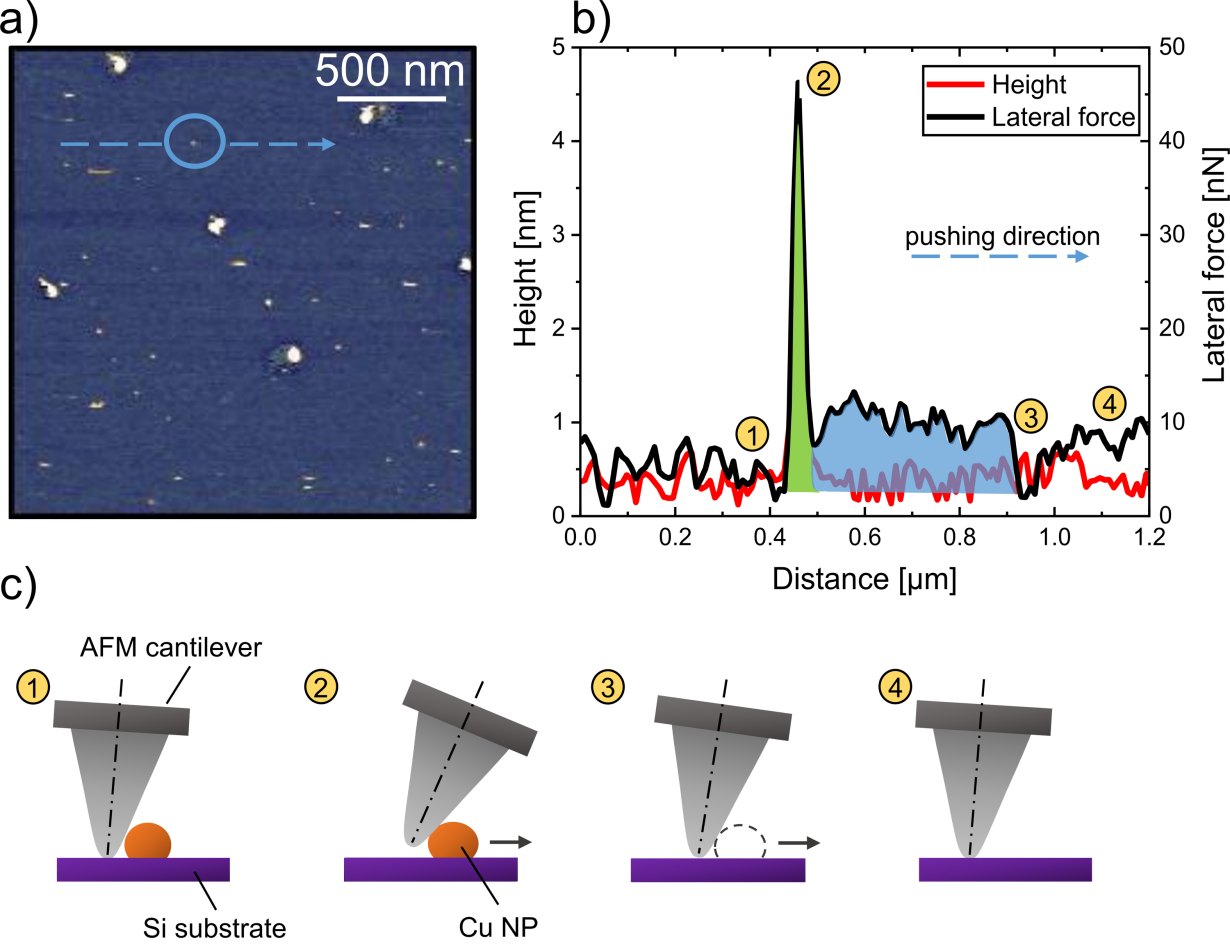
*(*a) Cropped AFM image of Cu NPs deposited at a bias voltage of 1000 V onto Si, *z*-scale of 14 nm. The blue arrow indicates the pushing direction of the chosen Cu NP and (b) the corresponding graph of the height and the determined lateral force over the distance (the green shaded area corresponds to the work of separation, whereas the blue area indicates the work needed to overcome friction). Points 1 to 4 indicate the cantilever movements in (c): (1) The tip approaches, but has not yet contacted, the NP; (2) the tip reaches the maximum force required to push the NP; (3) the NP is moved, causing the cantilever to bend and the measured lateral force to drop to a minimum value; and (4) the tip returns to its original position, completing the pushing cycle.

To provide a reliable and accurate measure of the energy required to manipulate the NPs, the total work of manipulation (*W*_m_) was calculated by taking the integral of lateral force (LF) over the manipulation distance (*l*_m_) in *x*-direction. The procedure is illustrated in Figure 4. For each NP pushing event, *W*_m_ was calculated using Equation 1:


[1] $$W_{\text{m}}=\int_{0}^{l_{\text{m}}}\text{LF d}x.$$

The area under the lateral force versus manipulation distance curve, as indicated by points 1 to 3 in Figure 4b, corresponds to the total work of manipulation. It should be noted that the derived values for the total work of manipulation include both the actual work of separation *W*_sep_ and the dissipated work *W*_diss_, which does not contribute to the separation. The relationship can be expressed as:


[2] $$W_{\text{m}}=W_{\text{sep}}+W_{\text{diss}}.$$

NP motion on a surface may not necessarily proceed as an ideal lateral sliding path. Depending on the local contact geometry, the displacement may also include a rolling component. According to Tafazzoli et al., sliding motion sets in first and is dominant over rolling motion [48]. When the force exceeds a critical threshold, rolling and sliding can occur simultaneously. However, since the maximum forces remain below 40 nN, a rolling contribution is highly unlikely. In addition, the presented formalism would not change if rolling motion was present, as it can be expressed analogously by the product of normal force and (rolling) friction coefficient. Thus, the overall NP displacement is described as a translation, covering both sliding- and rolling-like contributions [16]. The work extracted from the lateral force–distance integral therefore represents the effective energy required to translate a NP on the surface. For simplicity, we calculate the work of manipulation as a first approximation for the work of adhesion as the details in the process of manipulation are rather complex including adhesion, static and dynamic friction, and humidity [49]. Due to the challenges in direct calculation of the dissipated work, the work of separation is approximated using the following equation:


[3] $$W_{\text{sep}}\approx \frac{\left[F_{\text{static}}-\left(F_{\text{cap}}\cdot \mu +F_{\text{n}}\cdot \mu \right)\right]\cdot l_{\text{s}}}{2},$$

where *F*_static_ is the maximum static lateral force, *F*_cap_ represents the capillary force, and *F*_n_ is the normal force applied by the cantilever. μ is the coefficient of friction and *l*_s_ the separation distance given by the distance between the first tip/NP contact and the position with the maximum lateral force needed to separate the NP from the substrate surface [50]. Both *F*_static_ and *F*_cap_ are typically very small in magnitude, contributing only minimally to the overall force. The factor 1/2 accounts for the relevant part of the lateral force vs distance curve, which is to a good approximation triangular (see green shaded part of Figure 4b.

An exemplary calculation is provided for the NP marked by the blue arrow in Figure 4a, where the corresponding lateral force and height profiles are plotted in Figure 4b. The separation distance *l*_s_ is the *x*-separation between points 1 and 2 indicated in Figure 4b. A corresponding scheme is provided in Figure 4c. For the sake of brevity, from now on, we will use the term “distance” synonymously with “separation distance” as used in Equation 3. In more detail, at point 1, the cantilever approaches the NP on the Si substrate. Point 2 corresponds to the maximum lateral force required to initiate NP displacement, as shown by the green-shaded area, which represents the work of separation. Beyond this, at points 3 and 4, the NP is already pushed out of the intimate contact with the surface [49] and, thus, has overcome the adhesion strength. The blue-shaded area represents the work dissipated due to sliding friction, which is not included in the calculation. The approximation of work of separation assumes a linear NP translation along the scan direction. Minor deviations from this ideal path may occur, but they remain within the scatter of the presented data.

**Figure 5:**
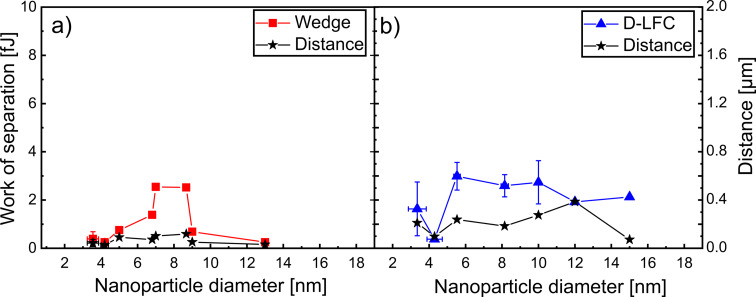
Work of separation as a function of NP diameter determined for Cu NPs deposited at a bias voltage of 0 V onto Si using (a) the wedge and (b) the D-LFC calibration methods.

While an increase in the work of separation with NP diameter was expected, the lateral force versus NP diameter in Figure 3b does not show a clearly increasing trend. Instead, the results presented in Figure 5 show a different behavior, as exemplified for a substrate bias voltage of 0 V. Specifically, both work of separation and distance reach maximum values for NP diameters between 6 and 10 nm. NPs outside this size range exhibited lower work of separation values as well as shorter distances. With an AFM tip diameter smaller than 10 nm, it has to be assumed that the behavior shown in Figure 5 is affected by the interaction between tip and NPs. This could also explain why both calibration methods show peak values at certain NP sizes, suggesting that each method has certain particle ranges where it performs most reliably.

**Figure 6:**
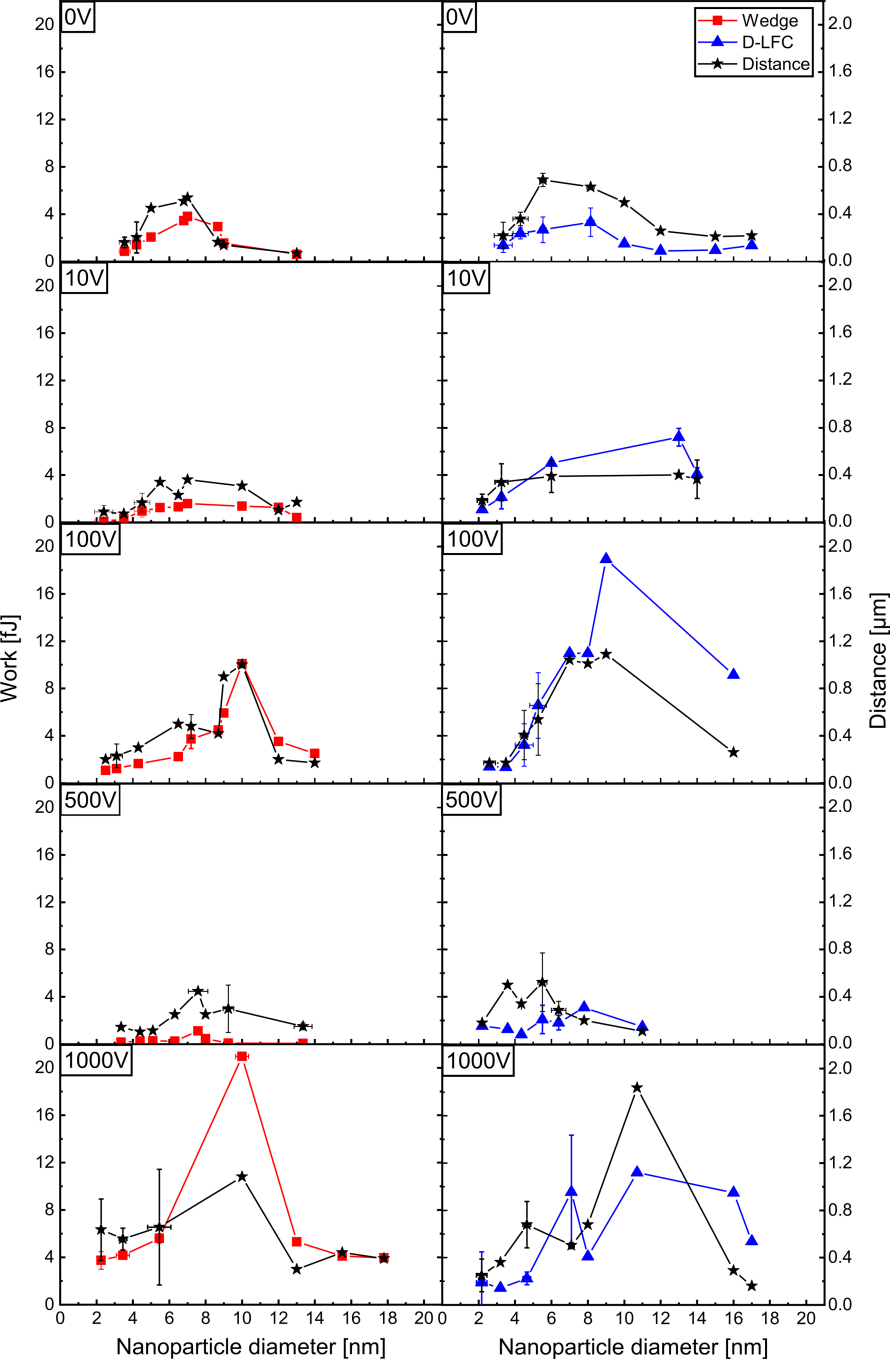
Relationship between work of separation and NP diameter at different substrate bias voltages. In addition, the distance (black stars) moved by the NPs is also indicated. For comparison, two sets of experiments with different lateral force calibration methods are shown (left column: wedge method; right column: D-LFC method). Each work of separation symbol represents the average of twelve data points. Note that some error bars are too small to be visible.

The relationship between work of separation and distance for the different NP diameter size ranges and substrate bias voltages is summarized in Figure 6. As the applied voltage increases, the peak values for both work of separation and distance shift from the 6–10 nm range to 10–12 nm. This indicates that a larger lateral force, and consequently a higher work of separation, is required to manipulate NPs in this size range. In our approach, the work of separation includes both the work of adhesion and the dissipated energy that is lost via different channels during the pushing experiment (e.g., deformation energy of NP and/or surface). Despite this, we consider the results to still reflect the work of adhesion between the NP and the surface reasonably well.

As generally expected, the typical work of adhesion increases with higher substrate bias voltage. For instance, although the total kinetic energy for 2 nm NPs increases by a factor of ≈390 between 0 and 1000 V bias, the energy per atom (≈2.8 eV·atom^−1^) is still clearly below the binding strength of a bulk Cu–Cu bond (≈3.5 eV·atom^−1^) [51]. This energy per atom remains near the upper limit of the soft-landing regime, but approaches the threshold of the intermediate regime [46]. Larger NPs (10–12 nm) require higher values of work of separation, as particularly evident at 100 and 1000 V. This may be due to increased adhesion, although interaction of the tip simultaneously with several NPs at once cannot be excluded.

Nevertheless, we observed a trend towards increasing overall translation distances (up to ≈2 µm at 1000 V) at higher acceleration voltages (Figure 6). Even though the work of separation invested to detach the NPs from their resting positions has no clear dependence on acceleration bias or NP size, the longer transition distances might be indicative for stronger NP/substrate adhesion. This is in line with the short translation distances and smallest work of separation values for the 500 V case.

Although the dataset shows considerable scatter, the size-dependent behavior can still be discussed on a qualitative level. In order to deliver a plausibility argument for our observation, we refer to the approach of Weir and McGavin [8], who developed an analytic model for describing the coefficient of restitution of NPs rebounding from an ideally flat and rigid surface. Note that it delivers just a semi-quantitative trend and is not a rigorous description of the process. Their model provides a criterion to determine whether an NP escapes or is captured upon impact. This condition is expressed as:


[4] $$\begin{array}{c} F_{0}>6\pi R\gamma \;(\text{NP escape)}\\ F_{0}<6\pi R\gamma \;(\text{NP capture),} \end{array}$$

where *F*_0_ is the force acting to detach the NP from the surface, and 6π*R*γ is the adhesive force trying to hold the NP on the surface. *R* represents the radius of curvature defining the NP contact region with the surface, and γ is the interface/surface energy. As previously discussed, the NP/substrate adhesion represents a complex system involving many parameters, most of which can only be approximated.

**Figure 7:**
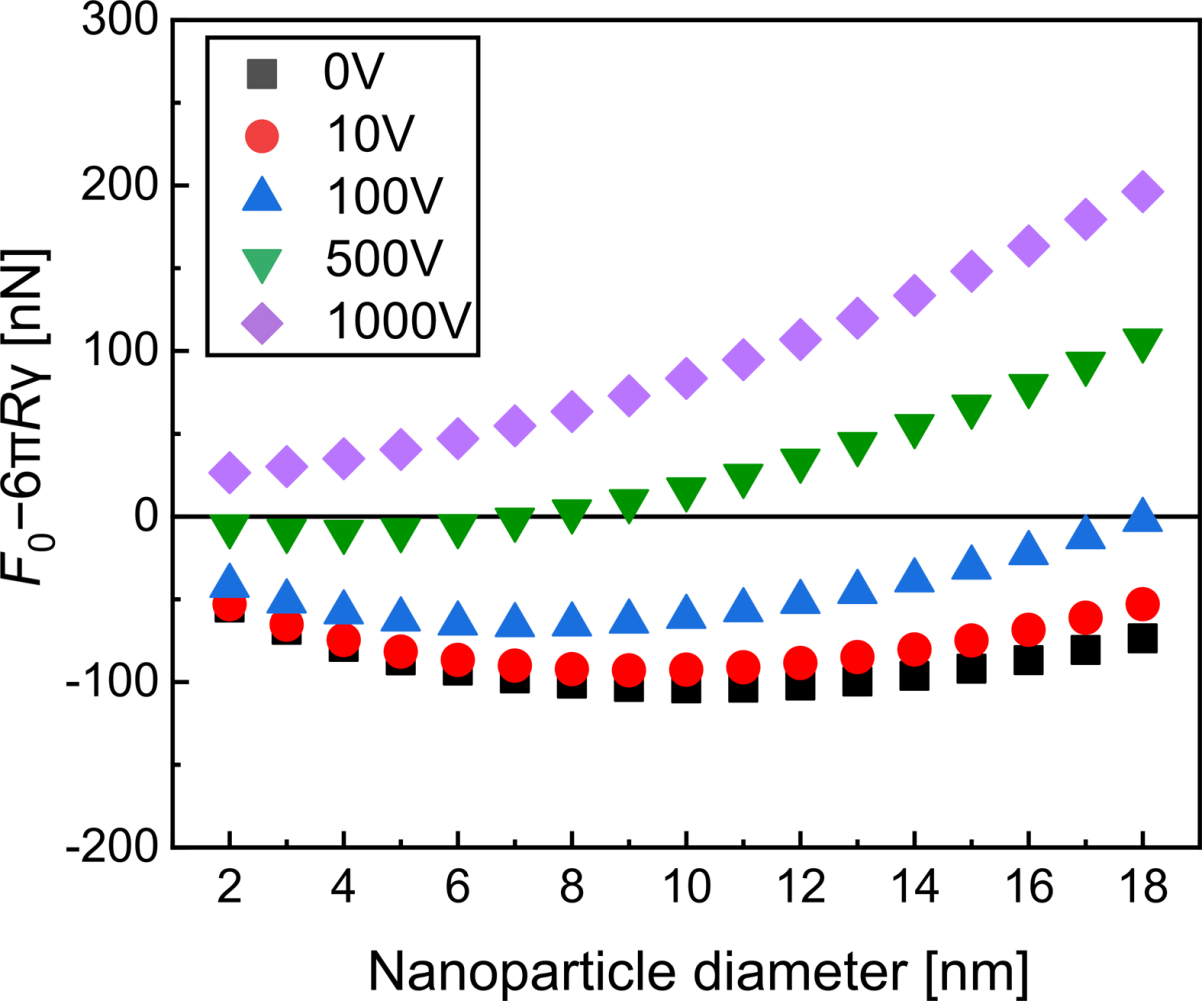
Deviation from the adhesion threshold *F*_0_ − 6π*R*γ as a function of NP diameter under varying substrate bias voltages.

For the calculation, the substrate is treated as ideally flat, perfectly smooth, and infinitely rigid, and its properties are therefore not considered in the model. The total velocity of a NP, *v_i_*, can be expressed as follows:


[5] $$v_{i}=A\cdot d^{-b}+\sqrt{\frac{12e\cdot V}{\rho \cdot \pi \cdot d^{3}},}$$

where the first term corresponds to the initial NP velocity as it exits the NP source orifice, and the second term is the velocity added due to the substrate bias *V*. The density ρ was assumed to be the room-temperature bulk density of Cu with 8935 kg·m^−3^ [52]. The parameters *A* and *b*, taken from the literature, are 188 m^1.35^·s^−1^ and 0.35, respectively [45], and *d* represents the NP diameter. NP charge states may vary as neutral, single, or multiple, while only at 0 V substrate bias of all them may arrive at the substrate. For simplicity, we assumed a single elementary charge *e* having a value of 1.6 × 10^−19^ A·s [53]. Choosing the mechanical properties of the NPs is not straightforward as their internal structure is unknown. Nevertheless, the following parameters were used for the model: γ was taken as 2.3 J·m^−2^ [54], the Young’s modulus *E* of copper as 150 MPa [4], and the yield strength *Y* of Cu NPs with a diameter of ≈20 nm as 11 GPa [55]. While *E* and *Y* are not explicitly included in Equation 5, both parameters are essential for the model. *Y* affects the NP/substrate contact area and the plastic deformation, which determine *F*_0_ and whether the NP will rebound or stick to the substrate. A high *Y* limits deformation, reducing *F*_0_; a low *Y* increases deformation and adhesion. *E* is applied in the calculation of the elastic energy, which contributes to the deformation behavior model and the energy balance used to describe the NP/substrate interaction in the model. For a high *E*, more elastic energy is stored, promoting rebound, while at a low *E*, less energy is stored, favoring sticking. The values might seem high compared to bulk or polycrystalline Cu materials, but such values are common for single-crystalline Cu. In Figure 7 the theoretical adhesion threshold, *F*_0_ − 6π*R*γ, values are plotted as a function of the NP diameter. Negative values indicate the sticking regime, and positive values correspond to NP rebound (compare Equation 4). As shown in Figure 7, these calculated values reach their minimum between 6 and 10 nm across all voltages, suggesting a transition point in NP behavior. Notably, lower values of *F*_0_ − 6π*R*γ mean a higher probability of sticking. This theoretical behavior matches well with our experimental observations, particularly with the stronger adhesion measured for particles in the 6–12 nm range with increasing substrate bias.

Interestingly, the model predicts a rebound for all NP diameters at a substrate bias voltage of 1000 V, which appears rather counterintuitive. This result stems from the oversimplifications within the model. In reality, the NPs are unlikely to be perfectly spherical; they likely possess facetted surfaces due to their crystalline nature. Consequently, both the interface energy and the mechanical properties can vary depending on the particle orientation and contacting facet, such as (001) and (111), as shown in [4]. Further, the yield strength effects are also entirely omitted. In addition, neither chemical interactions with the substrate nor its mechanical response are considered. For small NP diameters (2–4 nm), the predicted deformation seems clearly overestimated, as the theoretical contact radius exceeds the NP size, suggesting full flattening of the particle into a disk, which was not observed experimentally. However, it should be noted that the AFM tip radius was 7–10 nm, meaning that features smaller than this remain unresolved, and the true NP shape remains unknown.

An attempt to recalculate the specific interface energies from the measured work of adhesion, using theoretically calculated contact areas, yields values in the range of 6 ± 2 J·m^−2^ at 500 V to 30 ± 30 J·m^−2^ at 0–100 V and 150 ± 150 J·m^−2^ at 1000 V. The deviation from the input value of 2.3 J·m^2^ indicates the limitations of the model in capturing the full complexity of the interaction. While the case from 0 to 500 V is in fair agreement with interface energies of sputter-deposited Cu films grown on SiO_2_/Si substrates at temperatures of 100–120 °C [56], the 1000 V case deviates substantially. This is most likely to due to the high impact velocities, which also include a rapid and strong temperature increase upon impact. Thus, the validity range of the Weir and McGavin model, which does not include temperature effects, is exceeded. Such effects could include massive dislocation activity at the interface and formation of the Cu_3_Si intermetallic phase, both leading to interface strengthening. Future research on local interface formation between substrates and NPs with different kinetic energies would be necessary to establish a comprehensive understanding of NP adhesion. Despite the deviations between theoretically predicted and measured adhesion energies, the model still highlights a key point: A higher impact energy does not necessarily lead to stronger NP adhesion.

## Conclusion

In this study, we examined the adhesion properties of Cu nanoparticles (NPs) on Si substrates, with a focus on varying landing conditions affected by the applied substrate bias voltages during NP deposition. The examined NP sizes ranged from 1 to 18 nm. AFM was utilized to measure the lateral forces required for NP manipulation, and we explored both lateral force–distance curves and work of separation as characteristic values to evaluate NP adhesion. Lateral force–distance curves alone did not provide comprehensive understanding of NP/substrate adhesion, and no clear trends were observed when correlating lateral forces with bias voltages. In contrast, the work of separation calculated as the integral of lateral force over the distance offered more accurate and insightful characteristics for NP adhesion.

The proposed method highlights the interplay between NP landing conditions governed by deposition parameters and NP-specific values like their diameter, surface energy, Young’s modulus, yield strength, as well as their crystallography-related anisotropies. Higher bias voltages and increased NP sizes did not automatically result in stronger adhesion. Typically, the adhesion was the strongest for NP diameters between 6 and 12 nm and reduced for larger NPs. A simple analytical model showed qualitative agreement with the AFM-based results, confirming that the adhesion is not a monotonic function of the sample bias voltage.

AFM-based manipulation was shown to be a reliable and reproducible method for quantifying NP adhesion, yielding consistent results across different calibration methods. The observed relationship between deposition parameters and adhesion strength provides a practical framework for characterizing NP/substrate interactions. Such understanding is essential for developing reliable NP coatings, where adhesion directly influences coating stability, uniformity, and functional performance under varying environmental and mechanical stress conditions during the use of functional devices or surfaces. Future studies should focus on the effects of environmental factors, such as humidity and temperature, and the exploration of alternative NP/substrate combinations to expand the understanding of the adhesion mechanisms at the nanoscale.

## Supporting Information

File 1 AFM topography of manipulated Cu nanoparticles.

## Data Availability

Data generated and analyzed during this study is available from the corresponding author upon reasonable request.
