# Supplementary material for: Quantitative estimation of nanoparticle/substrate adhesion by atomic force microscopy
Source: Beilstein J Nanotechnol. 2026 Jan 2;17:1–14. doi: 10.3762/bjnano.17.1 (PMC12766167; doi:10.3762/bjnano.17.1)
Supplement: File 1 — AFM topography of manipulated Cu nanoparticles. [file Beilstein_J_Nanotechnol-17-01-s001.pdf]

BEILSTEIN JOURNAL OF NANOTECHNOLOGY

Supporting Information

for

Quantitative estimation of nanoparticle/substrate adhesion by atomic force microscopy

Aydan Çiçek, Markus Kratzer, Christian Teichert and Christian Mitterer

Beilstein J. Nanotechnol. **2026,** *17,* 1–14. doi:10.3762/bjnano.17.1

AFM topography of manipulated Cu nanoparticles

License and Terms: This is a supporting information file under the terms of the Creative Commons Attribution License (https://creativecommons.org/licenses/by/4.0). Please note that the reuse, redistribution and reproduction in particular requires that the author(s) and source are credited and that individual graphics may be subject to special legal provisions.
The license is subject to the *Beilstein Journal of Nanotechnology* terms and conditions: (https://www.beilstein-journals.org/bjnano/terms)

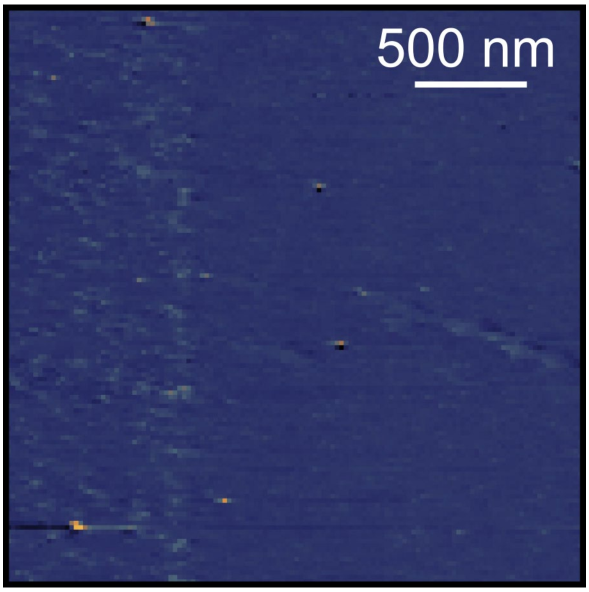

Figure S1: Cropped AFM topography image (5 × 5 μm^2) recorded on the same sample region as shown in Figure 2, *z*-scale of 14 nm. The image shows that displaced Cu NPs accumulate at the boundaries of the 1 × 1 μm^2 scan area used during manipulation. The central area corresponds to the effective pushing path of the AFM tip, while NPs outside this scan region remain in their initial positions.
